# Optimizing Process Parameters in Laser Transmission Welding Solid PC/Porous-PET Using Prediction Models: Experimental Validation and Morphology Analysis

**DOI:** 10.3390/ma19153177

**Published:** 2026-07-24

**Authors:** Jinqiang Li, Yitao Wu, Xiangsheng Luo, Siyu Zhou, Zijian Wang, Huang Zhang, Bowen Zhong, Haiyu Qiao

**Affiliations:** School of Mechanical and Electrical Engineering, Soochow University, Suzhou 215000, China

**Keywords:** laser transmission welding, machine learning, Gaussian process regression, process optimization, solid/porous polymer joining

## Abstract

Determining optimal process parameters for laser transmission welding (LTW) of solid/porous materials remains challenging due to the complexity of influencing factors. In this study, the welding of solid polycarbonate (PC) and porous polyethylene terephthalate (porous-PET) was chosen as an exemplary case and the relationship between parameters and welding quality was established using a Gaussian process regression (GPR) model. First, the experimental dataset, comprising welding power, welding speed, PC thickness, and porous-PET density, is established based on a flexible factor-level design. Then, the optimized GPR model trained based on the full experimental dataset achieved high predictive performance, significantly outperforming that trained with the averaged experimental dataset. Next, using the optimal prediction model as the objective function, three different optimization methods, genetic algorithm (GA), Bayesian optimization (BO), and covariance matrix adaptation evolution strategy (CMA-ES), are employed to optimize the process parameters, and the performance of the different optimization algorithms shows that CMA-ES has demonstrated the fastest convergence and the shortest runtime, while still converging to the same recommended parameters as GA and BO. Experimental validation confirms the accuracy of the recommended parameters, with a low relative error. Morphological analysis confirms that the weld seam is uniformly formed at recommended parameters. The proposed strategy provides an efficient route for achieving high-performance LTW joints and shows strong potential for improving process efficiency and reducing manufacturing cost in solid/porous materials joining.

## 1. Introduction

Laser transmission welding (LTW), as a representative plastics welding technique, offers the advantages of small heat-affected zone, low residual stress, and high welding precision, and has a wide range of applications in the automotive, medical, and electronic industries [1,2,3]. During the welding process, the laser beam first penetrates the upper transparent material and is converted into thermal energy at the interface by the absorbent material; then the thermal energy heats the local materials to a molten state, where the molecular chains diffuse and entangle with each other; finally, these entangled molecular chains solidify to form a high-strength welded joint upon cooling [4]. It is clear that the welding quality of joints is highly sensitive to various process parameters that affect molecular chain entanglement, which include material properties (such as compatibility), characteristics of the samples to be welded (such as density and thickness), and welding parameters (such as welding power, welding speed, and clamping pressure) [4,5]. Traditional trial-and-error optimization methods, such as single-factor experiments [6], orthogonal experiments [7,8] and response surface methodology [1], are effective for one or two factors, but inefficient or even fail for more than three factors, or factors with different levels. Therefore, efficiently determining the optimal process window is of great significance for achieving stable and high-quality welded joints.

Machine learning has demonstrated significant advantages in process modeling and optimization in fields such as laser processing [9], additive manufacturing [10], as well as polymer materials processing [11], owing to its ability to learn complex nonlinear mappings between processing factors and results. These techniques also offer a new research pathway for parameter optimization in LTW. For example, Kumar et al. combined response surface methodology with particle swarm optimization and teaching-learning-based optimization to compare the performance of different optimization strategies in welding parameter optimization [12]. Mehrpouya et al. compared the performance of feedforward multilayer perceptrons and radial basis function networks in predicting plastic welding quality, finding that the feedforward network achieved higher prediction accuracy (97.37%) [13]. Acherjee et al. constructed a backpropagation neural network model in which welding power, welding speed, defocusing distance, and clamping pressure were chosen as inputs to accurately predict lap shear strength and weld width [14].

Moreover, the systematic integration of prediction models with advanced optimization algorithms holds promise for achieving efficient inverse optimization of welding parameters [14,15]. For example, Liu et al. developed a serial artificial neural network (ANN) model and integrated it with a Markov decision process (MDP), achieving superior optimization results with only 15.5% of the computational resources required by standard methods [16]. Acherjee et al. constructed a multi-objective optimization framework based on an artificial neural network (ANN) and a non-dominated sorting genetic algorithm (NSGA-II), enabling simultaneous optimization of welding quality for solid-to-solid polypropylene joints [17]. Jiang et al. proposed an ANN model based on the crow-wolf optimization algorithm (CWO), which realized intelligent hyperparameter tuning and high-precision prediction of weld width for solid PC and solid PMMA welded pairs [18]. However, most of the aforementioned studies concentrate on solid–solid material combinations, and research concerning solid–porous dissimilar material pairs is quite scarce. In addition, the high-precision process prediction models proposed in these works mostly adopt datasets composed of single experimental measurements or averaged experimental datasets. Conventional experiments require at least three replicate tests to eliminate errors induced by equipment variations, manual operation and environmental disturbances. Nevertheless, limited research has focused on the composition of datasets, especially the prediction difference resulting from adopting full experimental datasets rather than averaged data.

In this study, the welding of solid polycarbonate (PC) and porous polyethylene terephthalate (porous-PET) is chosen as an exemplary case to investigate how the welding dataset, comprising both full and averaged experimental datasets, affects the performance of modeling and optimization based on the Gaussian process regression (GPR) method. First, the experimental dataset is established based on a flexible factor-level design. Then, the GPR method is trained on different datasets, the full and averaged experimental datasets, to map the relationship between process parameters and breaking force. The performance of the models trained under these two input conditions is compared. Next, using the optimal prediction model as the objective function, three different optimization methods are employed to optimize the process parameters, and the performance of the different optimization algorithms is compared in terms of optimal value, stability, and convergence. Finally, experimental verification is conducted on the obtained recommended combination of process parameters.

## 2. Materials and Experimental Methods

### 2.1. Materials and Welding Setup

PC sheets were purchased from Guangdong Xin Zheng Jia New Material Technology Co., Ltd. (Dongguan, China), with dimensions of 10 mm × 30 mm and thicknesses of 0.3, 0.55, and 1.0 mm, respectively. Porous-PET sheets with densities of 0.15 g/cm^3^ and 0.175 g/cm^3^ were provided by Shanghai Xin Pin Fluid Equipment Co., Ltd. (Shanghai, China) and Nanjing Chuangbo Extrusion Equipment Co., Ltd. (Nanjing, China), respectively. The porous-PET samples were cut to dimensions of 10 mm × 30 mm × 1.5 mm. The ethanol (99%) used in the experiments was purchased from Shanghai Meryer Chemical Technology Co., Ltd. (Shanghai, China). The welding equipment used was an SW-22 direct semiconductor laser (3S Laser, Shanghai, China) with a wavelength of 1710 nm, and a spot diameter of 1 mm. During welding, the laser source remained stationary, while the samples were moved by an ABB robotic arm. The samples were clamped by a glass fixture, and the clamping pressure was maintained at 0.43 MPa using a pneumatic clamping device. All welding experiments were conducted in a laboratory environment at approximately 25 °C, while the humidity was not strictly controlled.

### 2.2. Experimental Design

The experimental dataset includes process parameters (input parameters) and breaking force of the welded joint (output). The input parameters consist of four variables: welding power, welding speed, PC thickness, and porous-PET density (specific experimental values are shown in Table 1). Before welding, all samples were cleaned with ethanol and water to remove surface contaminants and then dried in an oven at 45 °C for 30 min. After welding, tensile–shear tests were performed using a universal testing machine, and each set of parameter combinations was tested three or more times to ensure the accuracy of the experimental results.

### 2.3. Characterization

Tensile–shear tests were conducted using a TY8000A universal testing machine (Tian Yuan Testing Instruments, Yangzhou, China) with an accuracy class of 0.5 (force measurement uncertainty within ±0.5%), with the upper fixture moving upward at a constant tensile rate of 2 mm/min. The microstructure of the welded joints was observed using an EVO25 scanning electron microscope (Zeiss, Oberkochen, Germany). The samples were coated with platinum for 80 s at 8 mA using a sputter coater (Quorum Q150R ES, Laughton, UK) to avoid electrical charging effects before SEM observations.

## 3. Model Structure Design

**Figure 1:**
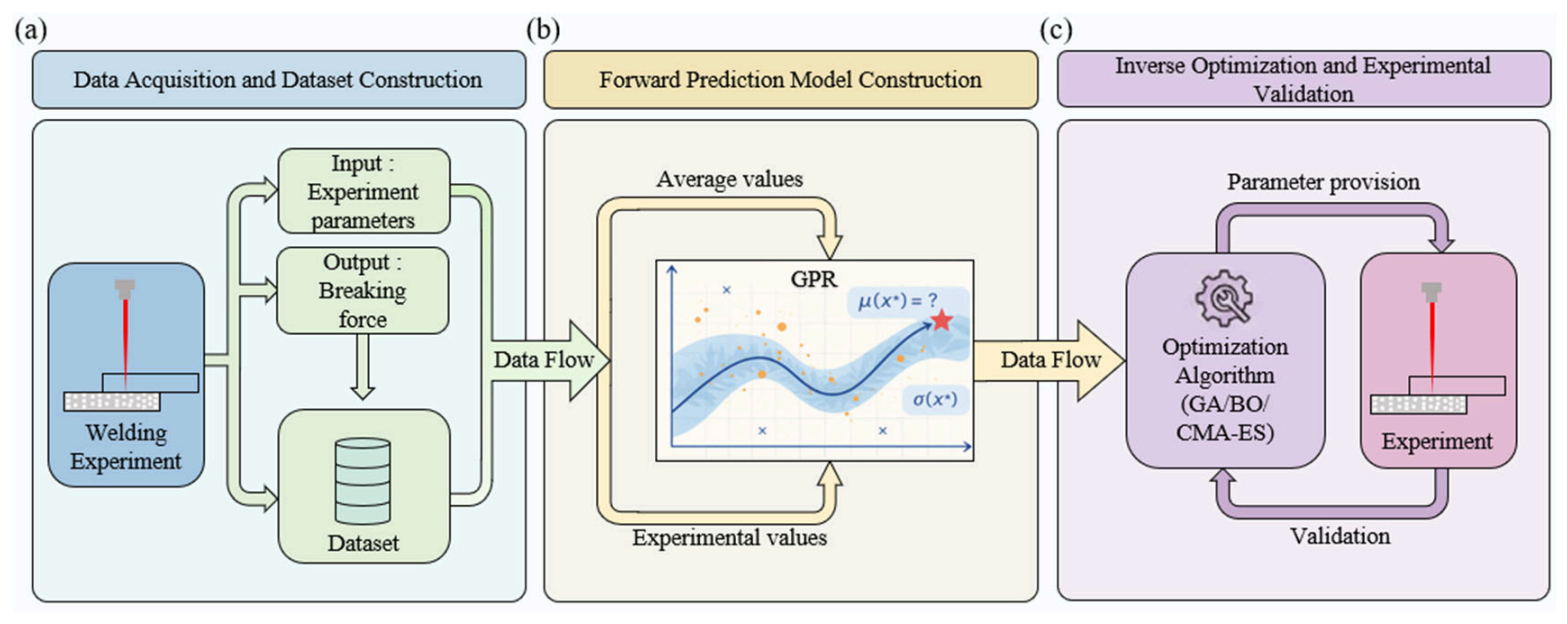
Proposed forward prediction and reverse optimization framework for laser transmission welding of PC/porous-PET using GPR. The framework consists of three core modules: (**a**) experimental dataset construction, (**b**) GPR-based forward prediction, and (**c**) reverse optimization and experimental validation.

Figure 1 presents the proposed forward prediction and reverse optimization framework for welding PC/porous-PET using LTW. The framework consists of three core modules: (a) Experimental dataset construction from welding experiments; (b) Forward prediction model using GPR, using both the full/averaged experimental dataset to map the relationship between welding process parameters and joint performance, where the effect of the dataset on the performance of the modeling is discussed; (c) Conduct optimization under specified conditions and perform experimental validation.

### 3.1. Experimental Dataset Pretreatment

To improve numerical stability and convergence efficiency of the model, Z-score standardization is applied to the input features and the target variable according to Equation (1):

(1) $$x_{\mathrm{scaled}}=\frac{x-\mu_{x}}{\sigma_{x}},\;y_{\mathrm{scaled}}=\frac{y-\mu_{y}}{\sigma_{y}}$$

where $\mu_{x}$ and $\sigma_{x}$ are the mean and standard deviation of each input feature in the training set, and $\mu_{y}$ and $\sigma_{y}$ are the mean and standard deviation of the target variable in the training set. After standardization, the data have $\mu_{x},\;\mu_{y}=0$, and $\sigma_{x},\;\sigma_{y}=1$.

### 3.2. Forward Prediction Model

#### 3.2.1. Gaussian Process Regression Model

Gaussian process regression is a non-parametric regression method based on the Bayesian framework [19]. Compared with an explicit specification of the network structure required in ANN, GPR implicitly defines a prior distribution over the function space through a kernel function, and is capable of simultaneously outputting both the predictive mean and the uncertainty estimate, making it suitable for nonlinear modeling tasks under small-sample conditions. The details of the principle of the GPR can be found in previous published papers [20]. In the GPR model, the objective function f(x) is assumed to follow a Gaussian process, which can be expressed as [21]:

(2) $$f(x)∼GP\left(m(x),k(x,x^{\prime })\right)$$

where m(x) is the mean function, set to zero in this paper; $k(x,x^{\prime })$ is the covariance function (kernel function), which measures the similarity between input points.

In this study, the RBF kernel function, the Matern kernel function, and the RQ kernel function were compared. The Matern kernel function was ultimately selected as the covariance function. Its smoothness is controlled by the parameter ν, which allows flexible adaptation to different characteristics of the data [22]. The hyperparameters of the kernel function (including signal variance, length scale, etc.) are adaptively optimized during model training by maximizing the marginal log-likelihood function.

The training of the GPR model is achieved by maximizing the marginal log-likelihood function. To avoid falling into local optima, a multi-start optimization strategy is adopted, setting multiple random initial points for multiple optimization restarts [21]. Meanwhile, to improve numerical stability and model robustness, a small noise term $\sigma_{n}^{2}$ is introduced to the diagonal of the kernel matrix to model the observation noise.

As a Bayesian non-parametric method, GPR prevents overfitting primarily through the following methods. First, kernel function selection and hyperparameter regularization: the study adopts the Matern kernel as the kernel function, whose hyperparameters (such as signal variance and length scale) are naturally constrained during the optimization process by the marginal log-likelihood function, thereby preventing the model from overfitting the training data [23]. Second, noise term introduction: adding a small noise term to the diagonal of the kernel matrix is equivalent to incorporating an assumption of observation noise into the model, enhancing the model’s robustness to random fluctuations in the training data [24].

For a new input point $x_{*}$, the GPR model provides the posterior distribution of the predicted value, with the predictive mean and predictive variance given by [21]:

(3) $$\hat{f}(x_{*})=k_{*}^{T}(K+\sigma_{n}^{2}I{)}^{-1}y$$

(4) $$Var[\hat{f}(x_{*})]=k(x_{*},x_{*})-k_{*}^{T}(K+\sigma_{n}^{2}I{)}^{-1}k_{*}$$

where K is the training set kernel matrix, $k_{*}$ is the covariance vector between the test point and the training set, and $\sigma_{n}^{2}$ is the noise term.

#### 3.2.2. Model Performance Evaluation

In this study, the root mean square error (RMSE) and the coefficient of determination (R^2^) are employed to evaluate the accuracy of the GPR models. RMSE measures the average magnitude of the prediction errors, and is defined as follows:

(5) $$RMSE=\sqrt{\frac{1}{m}\sum_{i=1}^{m}({\hat{y}}_{i}-y_{i}{)}^{2}}$$

where *ŷ_i_* and *y_i_* are predicted and experimental values, respectively. The smaller the RMSE, the better the model performance. R^2^ quantifies the proportion of variance in the dependent (target) variable that is explained by the independent variables in the model, and is defined as:

(6) $$R^{2}=1-\frac{\sum_{i=1}^{n}(y_{i}-{\hat{y}}_{i}{)}^{2}}{\sum_{i=1}^{n}(y_{i}-\bar{y}{)}^{2}}$$

(7) $$\mathrm{where}\;\bar{y}=\frac{1}{n}\sum_{i=1}^{n}y_{i}$$

The value of $R^{2}$ closer to 1 indicates a better fit of the model to the data.

### 3.3. Reverse Optimization Model

The reverse optimization module uses the forward prediction model trained in Section 3.2 as the objective function to search for the recommended parameter combination that provides high predicted breaking force within the given feasible range of process parameters. In this study, three heuristic optimization algorithms, namely genetic algorithm (GA), Bayesian optimization (BO), and covariance matrix adaptation evolution strategy (CMA-ES), are respectively employed for optimization, and the performance of each algorithm is systematically compared.

#### 3.3.1. Optimization Problem Definition

The optimization problem can be formally expressed as:

(8) $$\begin{array}{ccc} maximize\;x & & \hat{f}(x)\\ \mathrm{subject}\;\mathrm{to} & & x_{i}^{min}\leq x_{i}\leq x_{i}^{max},i=1,2,3,4 \end{array}$$

where $\hat{f}(x)$ is the predicted breaking force output by the forward prediction model; x=[P,v,t,ρ] represents the welding power, welding speed, PC thickness, and porous-PET density, respectively. This is a mixed-variable optimization problem: welding speed and welding power are continuous variables, while PC thickness (0.3, 0.55, 1.0 mm) and porous-PET density (0.15, 0.175 g/cm^3^) are discrete variables. The feasible range of each variable is determined by the parameter levels in the experimental design.

#### 3.3.2. Genetic Algorithm

Genetic algorithm (GA) is an evolutionary algorithm that simulates the biological mechanisms of natural selection and genetics, performing global search in the solution space through operations such as selection, crossover, and mutation. The principle of the GA can be found in previous published papers [25]. The key parameters of GA used in this paper are listed in Table 2.

#### 3.3.3. Bayesian Optimization

Bayesian optimization (BO) is a global optimization method based on a probabilistic surrogate model, particularly suitable for scenarios where the objective function is expensive to evaluate. Its core idea is to use existing observation data to construct a posterior distribution of the objective function and to guide the sampling of the next candidate point through an acquisition function. The principle of the BO can be found in previous published papers [26]. In this study, the kernel function of BO adopts a composite kernel (ConstantKernel + Matern + WhiteKernel), the acquisition function is EI (Expected Improvement), and other key parameters are listed in Table 3.

#### 3.3.4. Covariance Matrix Adaptation Evolution Strategy

Covariance matrix adaptation evolution strategy (CMA-ES) (based on Optuna 3.1.0) is a population-based evolutionary algorithm that leverages adaptive updates of the covariance matrix to learn the local geometric structure of the objective function. It offers the advantages of rotation invariance and insensitivity to the condition number. By simultaneously updating the mean vector and the covariance matrix, the algorithm adjusts its search direction, enabling more efficient exploration of complex high-dimensional optimization problems. In this implementation, the advantages of Optuna and CMA-ES are combined. The Optuna framework is used for parameter optimization, while the CMA-ES adaptively updates the covariance matrix and step size, thereby achieving efficient convergence in high-dimensional optimization problems. The principle of the CMA-ES can be found in previous published papers [27]. The key parameters in CMA-ES are listed in Table 4.

#### 3.3.5. Optimization Performance Evaluation

This study evaluates the optimization performance of the algorithms using the following dimensions: (1) accuracy: The magnitude of the optimal objective value (predicted breaking force) found by the algorithm; (2) convergence speed: The number of iterations required to reach a stable optimal solution; (3) robustness: The standard deviation of the optimal values obtained from multiple independent runs, reflecting the algorithm’s sensitivity to initial conditions. Each algorithm is run independently multiple times, recording the optimal value, convergence curve, and the number of iterations required to reach the optimal solution for each run (Table 5).

## 4. Results and Discussion

### 4.1. Experimental Data Distribution Analysis

**Figure 2:**
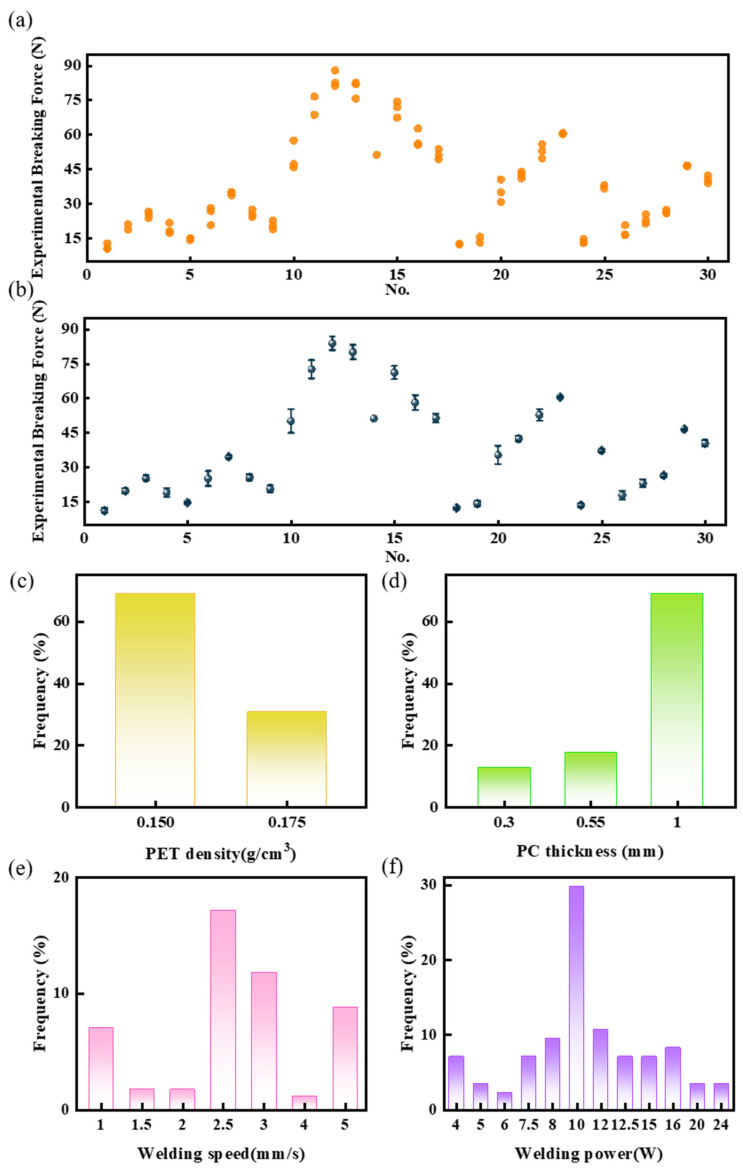
(**a**) Scatter plot of the full experimental dataset containing 84 data points. (**b**) Plot of the averaged experimental dataset, where error bars denote the standard deviation. Distribution analysis of experimental data. Frequency distributions of joints prepared under different processing parameters: (**c**) PET density, (**d**) PC thickness, (**e**) welding speed, (**f**) welding power.

The full experimental dataset, which contains a total of 30 process conditions with 84 records in total, is plotted in Figure 2a. It should be noted that points with the same x-coordinate represent repeated experiments under identical parameter conditions. The breaking force ranges from 10.28 to 87.90 N, indicating that the selected welding process parameters have a strong effect on the welding performance, consistent with previous reports [28]. In addition, different welding parameters can lead to similar welding performance, demonstrating that the effect of welding parameters is complex rather than a simplistic linear relationship [15]. Figure 2b further plots the averaged experimental dataset along with the variance under the same process states. It is clear that the variance is smaller (0–5.18874 N), falling into the repeatability variation reported in previous studies [29]. When using the full experimental dataset as training input, to avoid data leakage (i.e., three samples under the same experimental condition: some used for training, others for validation and testing, which would result in artificially high prediction performance), dataset partitioning follows the principle: any parameter combination present in the training set will not appear in the validation set or test set [30]. Figure 2c–f shows the frequency distributions of joints prepared under different processing parameters, including PET density, PC thickness, welding speed, and welding power. It can be clearly observed that the data exhibit an obvious imbalance. Owing to the data imbalance, the parameter combinations with fewer occurrences are first assigned to the training set. Then, in accordance with the aforementioned principle of avoiding data leakage, the remaining data are divided such that 65% are allocated to the training set, and the rest are randomly and equally split between the validation set and the test set.

### 4.2. Forward Prediction Model Performance

To better assess the effect of the experimental dataset on the prediction performance, the GPR model is first optimized and then compared.

#### 4.2.1. Optimization of GPR Model

**Figure 3:**
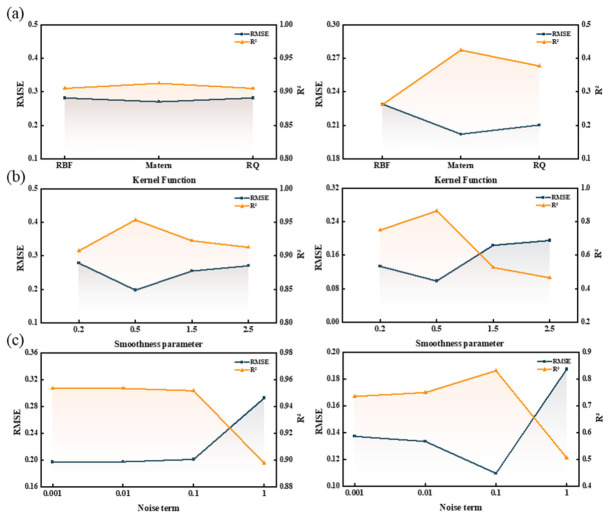
Optimizing GPR model Structure on different experimental dataset. (**a**) Three Different Kernel Functions. (**b**) Optimization of Smoothing parameter (v). (**c**) Optimization of Noise term $\left({\;\sigma }_{n}^{2}\right)$. The left/right column represent the full and averaged experimental datasets, respectively.

The kernel function type, smooth parameter (v) and noise term (${\;\sigma }_{n}^{2}$) all determine the performance of the GPR model. After determining these three key settings, this paper further optimizes and trains the hyperparameters within the kernel function (signal variance, length scale), ultimately obtaining the optimized GPR model. To ensure fairness and statistical reliability in the hyperparameter comparison, the one-factor-at-a-time method is adopted to guide the optimization process. That is, during the kernel function selection stage, other parameters are fixed, and only the kernel function type is varied. After the kernel function is selected, the remaining hyperparameters are optimized step by step to ensure the reliability of the comparison results at each stage. It should be noted that for the model trained on the averaged experimental dataset, both validation and testing used averaged values rather than individual measurements. Figure 3 shows the hyperparameter optimization process of the GPR model under the two input approaches, where the left subfigures correspond to the full experimental dataset input and the right subfigures correspond to the averaged experimental dataset input. Figure 3a compares the RMSE and R^2^ of three kernel functions (RBF, Matern, RQ) on the validation set. It can be seen that, regardless of whether the full experimental dataset or the averaged experimental dataset is used as input, the Matern kernel achieves the best prediction performance. Therefore, this study selects the Matern kernel as the covariance function for GPR.

After selecting the kernel function, the smoothness parameter ν of the Matern kernel is further optimized. As shown in Figure 3b, when ν is too small or too large, the model performance degrades, indicating an optimal range of smoothness. Considering both RMSE and R^2^, ν = 0.5 is determined as the optimal value.

After fixing the kernel function and ν, the noise term $\sigma_{n}^{2}$ is optimized. Figure 3c shows the model performance under different $\sigma_{n}^{2}$ values. Notably, the trends of noise optimization differ significantly between the two input approaches. When the full experimental dataset is used as input,${\;\sigma }_{n}^{2}$ = 0.01 achieves the best prediction performance, and further increasing the noise leads to a decline in fitting ability. In contrast, when the averaged experimental dataset is used as input, the model performance continuously improves as ${\;\sigma }_{n}^{2}$ increases from 0.001 to 0.1, with the optimal point at ${\;\sigma }_{n}^{2}$ = 0.1. The reason for this difference is that the averaged input eliminates intra-group experimental variability, leaving almost no noise in the training set, which makes the model prone to overfitting. Appropriately increasing the noise term (${\;\sigma }_{n}^{2}$ = 0.1) introduces regularization, effectively suppressing overfitting and thus improving generalization ability. In comparison, the full experimental dataset inherently contains intra-group fluctuations, so the model already possesses some noise robustness, requiring only a small noise term (${\;\sigma }_{n}^{2}$ = 0.01) to achieve optimal performance. Therefore, it can be seen that the full experimental dataset preserves intra-group variability, which provides useful information for improving the fitting performance of the GPR model.

Comparing the overall fitting performance under the two input approaches, the GPR model trained with the full experimental dataset significantly outperforms that using the averaged experimental dataset in all metrics. This is because the averaged input loses intra-group variation information, making it difficult for the model to learn the true data distribution and increasing the risk of overfitting. Therefore, the subsequent study adopts the GPR model trained with the full experimental dataset as the forward prediction model. The final kernel function configuration and hyperparameters of GPR are summarized in Table 6.

**Figure 4:**
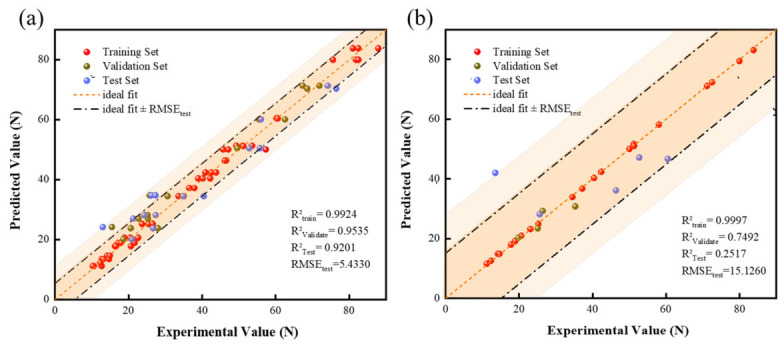
Comparison of prediction performance between models trained on full and averaged experimental datasets. (**a**) Model based on the full experimental dataset. (**b**) Model based on the averaged experimental dataset. The orange dashed line represents the ideal fit line (y = x), and the dark shaded area represents the range of the ideal fit line ± the unnormalized RMSE of the test set.

To evaluate the prediction performance of GPR models under two different input types (full experimental dataset vs. averaged experimental dataset), scatter plots of predicted versus experimental breaking forces from the training, validation, and test sets, are shown in Figure 4. Figure 4a presents the fitting performance of the GPR model trained with full experimental values. The scatter points for all three datasets are tightly distributed around the ideal fit line (y = x), indicating that the model successfully captures the nonlinear mapping from process parameters to breaking force. The test set achieves R^2^ = 0.9201 and unnormalized RMSE = 5.4330 N, which is used here for a clear illustration of the fitting performance in Figure 4, while all other RMSE values reported in this paper are normalized, demonstrating good generalization capability. In contrast, Figure 4b shows the results when the model is trained with mean values of breaking force per parameter combination. The training set points lie almost exactly on the ideal fit line, indicating nearly perfect memorization (overfitting). However, the validation and test set points deviate significantly from the ideal line, with much larger prediction errors. The test set R^2^ drops to 0.2517 and unnormalized RMSE rises to 15.1260 N. This severe overfitting occurs because averaging removes within-group variability, yielding training targets that are unnaturally smooth. Although the GPR model can perfectly fit such noise-free data, the intra-group variability differs across parameter combinations, and thus the averaged values for each condition exhibit different relative offsets after averaging. As a result, the trend learned from the training set is unlikely to generalize to the averaged trends of other datasets, and the model only captures the specific averaged pattern of the training set. Therefore, the GPR model trained with the full experimental dataset is selected as the surrogate function for subsequent inverse optimization.

To further investigate the superiority of the GPR model, using the full experimental dataset as input, the optimized GPR model and the optimized ANN model were independently run ten times each. The RMSE and R^2^ on the test set, as well as the single prediction time under the same parameter combination (porous-PET density 0.15 g/cm^3^, PC thickness 1.00 mm, welding speed 1.00 mm/s, welding power 10 W), were recorded. The results are summarized in Table 7.

As can be seen from Table 7, GPR achieved exactly the same performance across all ten runs (RMSE = 0.2477, R^2^ = 0.9201), while ANN showed fluctuations in RMSE between 0.2485 and 0.2957 and in R^2^ between 0.8861 and 0.9195, with an average RMSE of 0.2665 and an average R^2^ of 0.9072. This indicates that GPR, as a Bayesian nonparametric method, possesses better robustness and repeatability. More importantly, the single prediction time of GPR is only 0.428 ms, whereas ANN requires an average of 73.603 ms. The prediction speed of GPR is approximately two orders of magnitude faster than that of ANN.

The significant speed advantage of GPR over ANN stems from the fundamental difference in their prediction processes: GPR prediction involves only analytical calculations of the kernel function, i.e., the result is obtained through a linear combination based on the inverse of the training kernel matrix and the test covariance vector, without requiring layer-by-layer forward propagation through a multi-layer network [31]. Moreover, this dataset contains only 84 experimental data points, which is a typical small-sample scenario. Under such conditions, GPR can fully exploit the kernel function to capture nonlinear relationships in the limited data, whereas ANN is prone to overfitting the training data and its expressive power from the multi-layer structure cannot be fully utilized. Therefore, GPR not only outperforms ANN in prediction accuracy for small samples, but its extremely fast single-prediction speed also brings significant advantages for subsequent inverse optimization. In GA, BO, and CMA-ES, the objective function needs to be called tens of thousands of times; using GPR as the surrogate model can greatly reduce the total optimization time, making it possible to achieve efficient inverse optimization under limited experimental data.

To verify that the superior performance of the full-dataset model is not dependent on a specific data split, we repeated the splitting process using five different random seeds (42, 32, 24, 16, 8) based on the optimized model parameters. As shown in Table 8, the model achieved consistently high predictive performance across all five validation splits, with an average validation R^2^ of 0.9342 and an average RMSE of 0.2123. The small variation across the five splits confirms that the model performance is robust and not an artifact of a particular data division. In comparison, under the same five random seed splits, ANN achieved an average R^2^ of 0.9169 and an average RMSE of 0.2374, while GPR consistently obtained lower RMSE and higher R^2^ across all splits, indicating that GPR stably outperforms ANN in prediction accuracy regardless of the data split.

**Figure 5:**
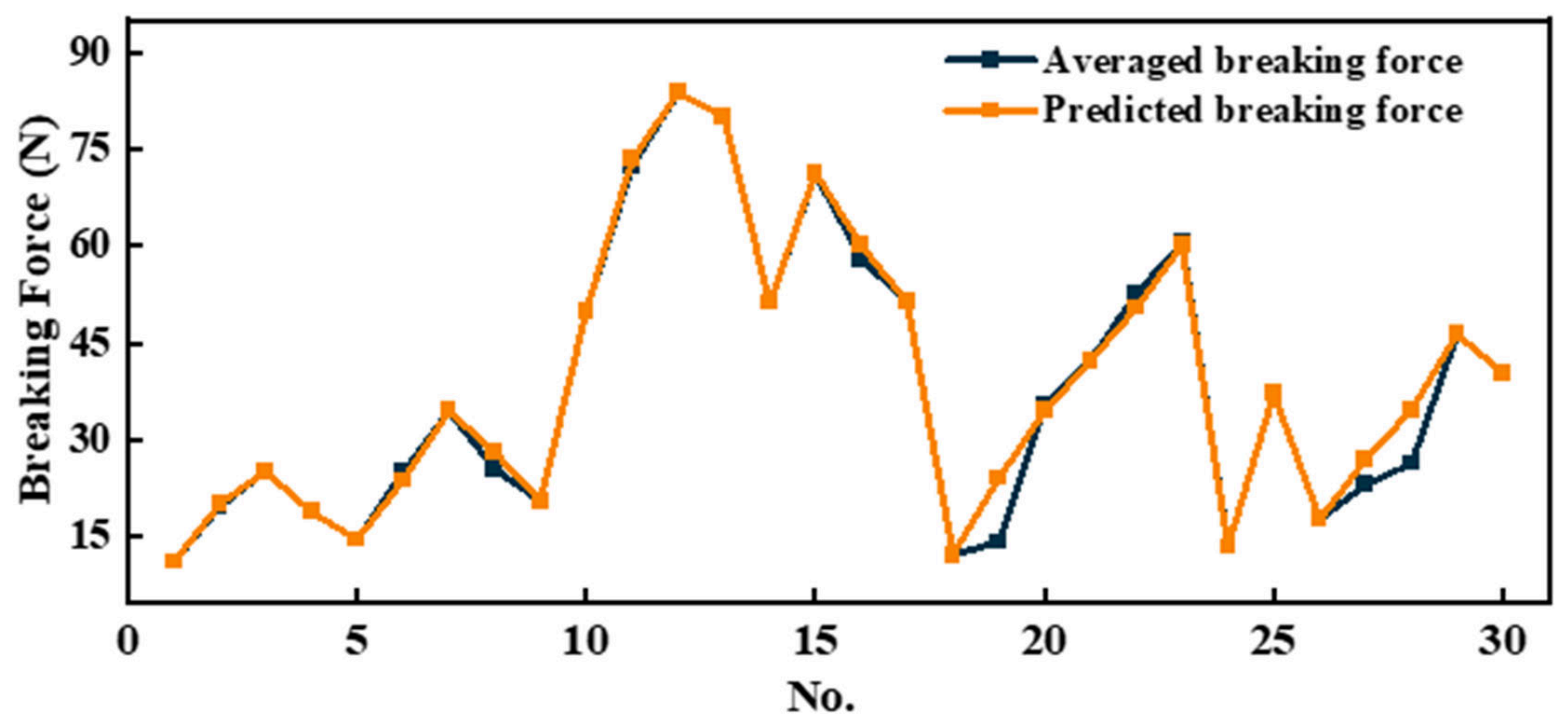
Prediction performance of the GPR model on the full experimental dataset. The dark blue line represents the mean experimental breaking force for each parameter combination, and the orange line represents the GPR predicted values. The parameter combinations are arranged sequentially along the horizontal axis.

To more intuitively demonstrate the prediction performance of the GPR model using the full experimental dataset as input on the entire dataset, Figure 5 presents the experimental average breaking force and the GPR predicted values for each parameter combination. The dark blue line represents the mean breaking force for each parameter combination; the orange line represents the GPR predicted values. The process parameter combinations are arranged sequentially along the horizontal axis. As can be seen from Figure 5, the predicted values are highly consistent with the trend of the experimental means, with only very few conditions showing large deviations. This further indicates that the GPR model using the full experimental dataset as input can effectively capture the intrinsic mapping relationship between process parameters and breaking force, and can provide reliable prediction results even for parameter combinations with large experimental fluctuations.

#### 4.2.2. Sobol Sensitivity Analysis

To quantitatively evaluate the influence of each process parameter on the breaking force and to provide a physical interpretation for the GPR model, this study employs the Sobol global sensitivity analysis method. An introduction to Sobol sensitivity analysis can be found in previously published papers [32], and an intuitive representation can be found in previous publications [33]. A specific Python library, SALib 1.4.7, was used to calculate the first-order, second-order, and total-effect Sobol indices for the continuous welding parameters. Note that although PC thickness was tested at only three discrete levels in the experiments, it is treated as a continuous variable in the Sobol analysis to assess its relative sensitivity within the experimental range, as the analysis aims to provide trend-level guidance rather than predictions at unmeasured thickness values.

For a model output $Y={f(x}_{1},x_{2},\ldots ,x_{k})$ the total variance V(Y) can be decomposed as [34]:

(9) $$V\left(Y\right)=\sum_{i}V_{i}+\sum_{i<j}V_{ij}+\cdots +V_{12\ldots k}$$

where $V_{i}$ is the first-order variance contribution of parameter $x_{i}$ acting alone, and $V_{ij}$ is the second-order interaction contribution between $x_{i}$ and $x_{j}$. The first-order sensitivity index $S_{i}=V_{i}/V\left(Y\right)$ measures the direct effect of $x_{i}$, while the difference between the total-effect index ($S_{T_{i}}$) and the first-order index ($S_{i}$) indicates the presence of interactions and higher-order effects.

Since porous-PET density is a discrete variable (only 0.15 and 0.175 g/cm^3^), it is fixed at the level with higher frequency (0.15 g/cm^3^). The analysis is performed only on the three continuous variables: PC thickness, welding speed, and welding power. Using the trained GPR model as the surrogate model, samples are generated via the Saltelli sampling method with a base sample size of N = 1024, and the Sobol indices are calculated using the SALib library.

**Figure 6:**
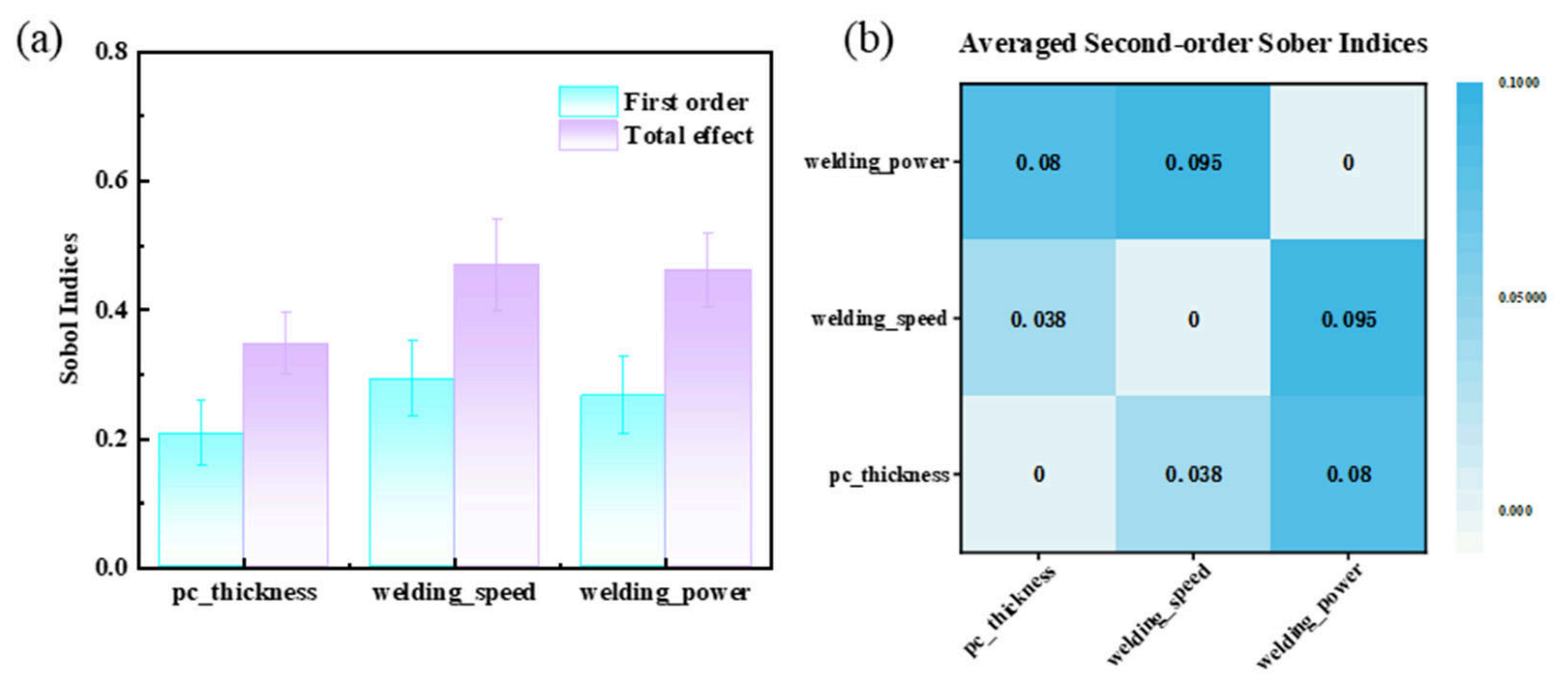
Sobol global sensitivity analysis results. (**a**) Bar chart of first-order and total-effect sensitivity indices with standard deviation error bars, reflecting individual and comprehensive parameter contributions to breaking force. (**b**) Heatmap of pairwise second-order interaction indices, quantifying the synergistic coupling strength between every two parameters.

Figure 6a presents the first-order and total-effect Sobol indices plotted as bar charts with error bars (±standard deviation). Welding speed exhibits the highest total-effect index (0.4693), followed by welding power (0.4608), indicating that these are the dominant factors. PC thickness has a lower total-effect index (0.3468) but still contributes to some extent. Among the first-order indices, welding speed (0.2918) and welding power (0.2661) are the largest, while PC thickness (0.2066) is smaller. The differences between total-effect and first-order indices for welding speed and welding power are 0.1775 and 0.1947, respectively, indicating the presence of notable interactions and higher-order effects involving these two parameters. Their pairwise interaction strengths are directly quantified by the second-order indices ($S_{ij}$ = 0.095 for welding speed and welding power) in Figure 6b.

Figure 6b presents the second-order interaction indices $S_{ij}$. The interaction between welding speed and welding power is the strongest ($S_{ij}=0.095$), confirming a significant synergistic effect between the two. The interaction between welding power and PC thickness is moderate (0.080), while the interaction between welding speed and PC thickness is weaker (0.038).

The above results are consistent with the physical mechanism of laser transmission welding: welding speed and power jointly determine the linear energy density, thereby controlling the interfacial heating and bonding quality. The strong interaction between them indicates that they need to be adjusted synergistically rather than independently during optimization. This analysis verifies the interpretability of the GPR model and provides guidance for subsequent reverse optimization: prioritize the matching of welding speed and power, followed by PC thickness.

### 4.3. Reverse Optimization Results from Different Optimization Algorithms

After completing the construction and selection of the forward prediction model, this section uses the GPR model trained with the selected full experimental dataset as the surrogate function, and employs three heuristic optimization algorithms: GA, BO and CMA-ES, to identify a recommended process window with high predicted breaking force within the given feasible range of process parameters. All optimization experiments are conducted under the condition of a fixed porous-PET thickness of 1.5 mm, consistent with the design of the reverse validation experiments.

To verify the effectiveness of the optimized process parameters, reverse optimization laser welding experiments were conducted under the condition of a fixed porous-PET thickness of 1.5 mm. To objectively compare the optimization performance of the three algorithms, each algorithm was independently run 20 times, and the optimal value, the number of iterations required to reach the optimal solution, and the runtime were recorded for each run. Table 9 summarizes the recommended parameter combinations obtained from a single typical run for the three algorithms, along with their corresponding predicted breaking forces. As can be seen from Table 9, all three algorithms converged to almost the same recommended parameter combination: porous-PET density of 0.15 g/cm^3^, PC thickness of 1.00 mm, welding speed of 1.00 mm/s, and welding power of approximately 10.55 W, with predicted breaking forces all reaching 82.26 N. This indicates that the selected GPR surrogate function exhibits good convexity within this parameter space, and all algorithms were able to effectively locate the same high-performance region.

In terms of computational efficiency, CMA-ES had the shortest average runtime (2.69 s), followed by GA (6.32 s), while BO had the longest runtime (14.47 s). The longer runtime of BO is mainly attributed to the need to refit the Gaussian process surrogate model at each iteration, whereas CMA-ES and GA only require simple forward prediction calculations. Notably, due to the inherently high computational efficiency of the GPR prediction model, the total runtime of all three algorithms was significantly lower than when ANN was used as the surrogate model.

In terms of convergence speed, according to the algorithm run records, CMA-ES achieved a stable optimal solution within approximately 500 iterations, with a smooth convergence process. BO rapidly improved the objective value during the initial sampling stage, followed by a fine-tuning search phase. Due to its population-based evolutionary mechanism, the convergence curve of GA exhibited a step-like pattern, but its early stopping mechanism effectively avoided unnecessary computational overhead. In terms of robustness, the standard deviations of the three algorithms were all very small, demonstrating good stability.

It is worth noting that the predicted breaking force at the recommended condition (≈82 N) is slightly lower than the single highest measured value in the training set (87.90 N), which was recorded at (1 mm/s, 10 W). Further discussion will be provided in Section 4.4.3.

Considering the four dimensions of convergence speed, optimization accuracy, robustness, and computational efficiency comprehensively, CMA-ES demonstrated the best overall performance in this study, achieving the fastest convergence and the shortest runtime, while giving a similar recommended process window to GA and BO. The above optimization results will serve as the input parameters for subsequent experimental validation, and the engineering reliability of the predicted recommended condition will be verified through actual welding experiments.

**Figure 7:**
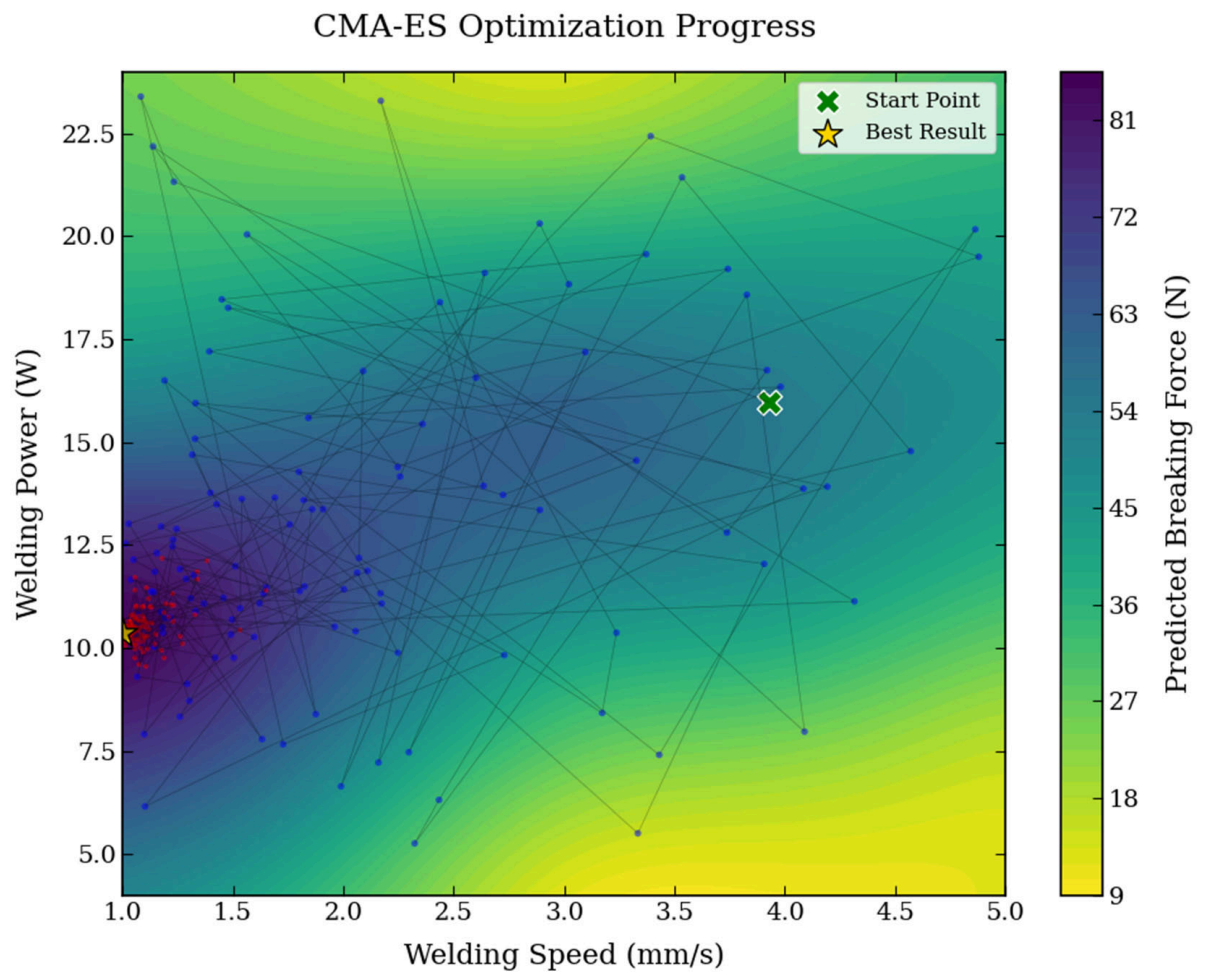
Optimization evolution of welding speed and welding power using CMA-ES. Blue dots represent the global exploration stage in the first 120 iterations, while red dots denote the local exploitation stage.

To further illustrate the searching behavior of CMA-ES, Figure 7 visualizes the evolution of welding speed and welding power during the optimization process. The blue dots represent the first 120 iterations (exploration stage), while the red dots denote the subsequent iterations (exploitation stage). As observed, the blue dots are widely scattered across the parameter space, indicating that CMA-ES thoroughly explores the feasible region in the early stage. In contrast, the red dots gradually converge and concentrate in a small high-performance region near the optimum (welding speed ≈ 1.0 mm/s, welding power ≈ 10.55 W). This demonstrates that CMA-ES (based on Optuna) not only achieves good global exploration but also maintains efficient local exploitation, which contributes to its fast convergence and high robustness.

### 4.4. Experimental Validation

#### 4.4.1. Experimental Setup and Sample Morphology

To verify the engineering reliability of the recommended process parameters obtained from the reverse optimization, a validation experiment was conducted using the recommended parameter combination (porous-PET density: 0.15 g/cm^3^, PC thickness: 1.00 mm, welding speed: 1.00 mm/s, welding power: 11 W, rounded from 10.55 W).

**Figure 8:**
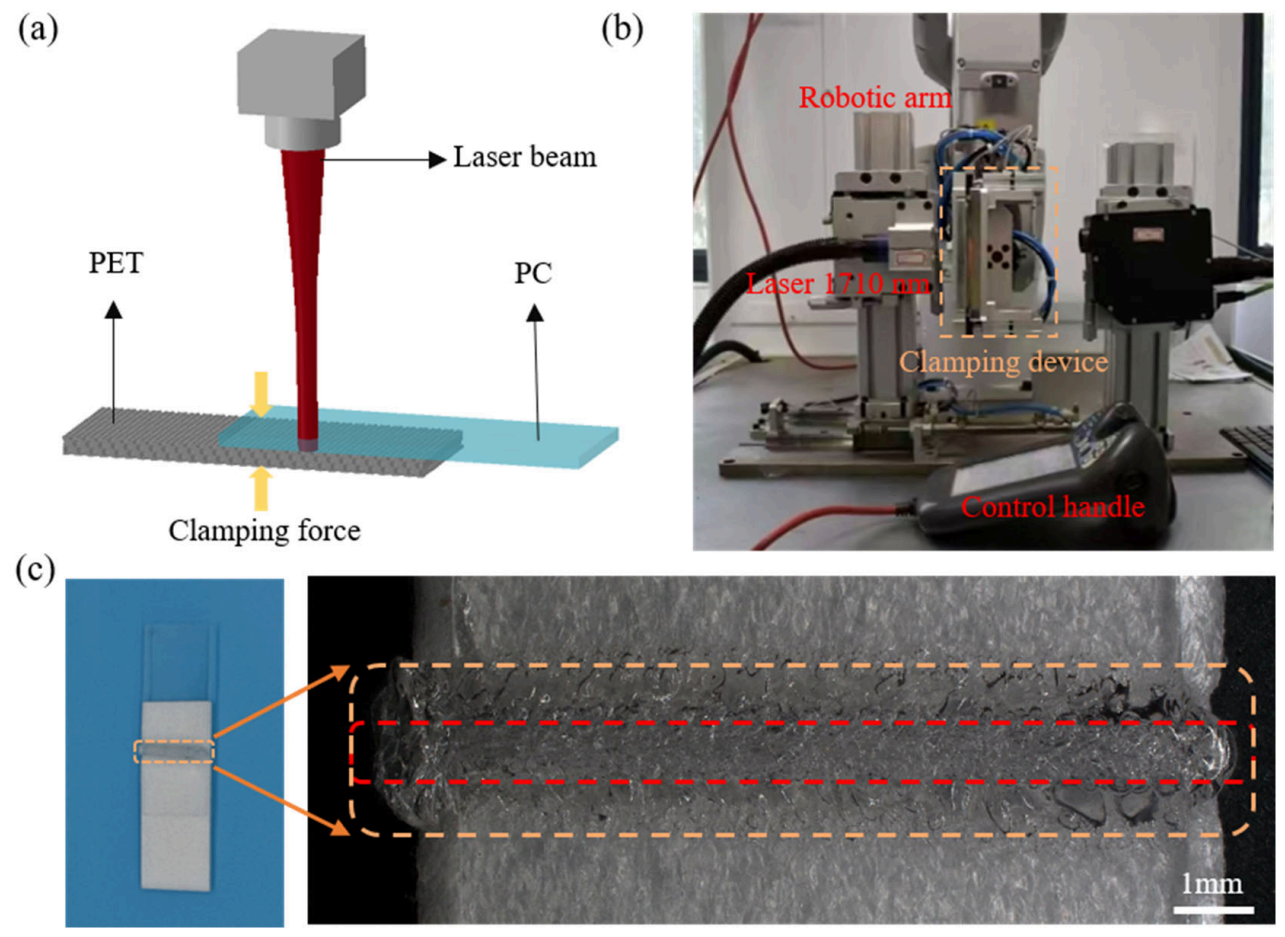
Experimental setup and weld morphology. (**a**) Schematic diagram. (**b**) The LTW setup using the 1710 nm laser. (**c**) Depth-of-field magnified image of the weld morphology.

Figure 8 illustrates the experimental apparatus and the morphology of the welded sample. Figure 8a shows a schematic diagram of the laser transmission welding setup, where a 1710 nm laser beam passes through the PC sheet and is absorbed at the PC/porous-PET interface. The laser head is mounted on a robotic arm for precise motion control. Figure 8b displays the actual 1710 nm laser device used in the experiments. Figure 8c presents a depth-of-field magnified image of the weld morphology. The region within the red dashed box indicates a fully fused weld seam that was formed perfectly without visible defects. Outside the red box but inside the orange dashed box, some bubbles can be observed. These bubbles were observed in the peripheral region outside the fully fused weld seam. While their exact origin remains to be clarified, a plausible explanation is the volatilization of residual substances on the porous-PET surface during laser irradiation. Further investigation would be required to confirm the underlying mechanism. It is also noted that the weld width is difficult to determine accurately, even under depth-of-field magnification. This is the reason why the breaking force, rather than the shear strength, is adopted as the evaluation metric in this study, and this practice has also been used in previous studies [34]. However, it should be emphasized that the breaking force is not equivalent to the shear strength, since the latter is a normalized quantity per unit area, whereas the former depends on the joint geometry and the effective bonded area.

#### 4.4.2. Mechanical Performance and Fracture Analysis

**Figure 9:**
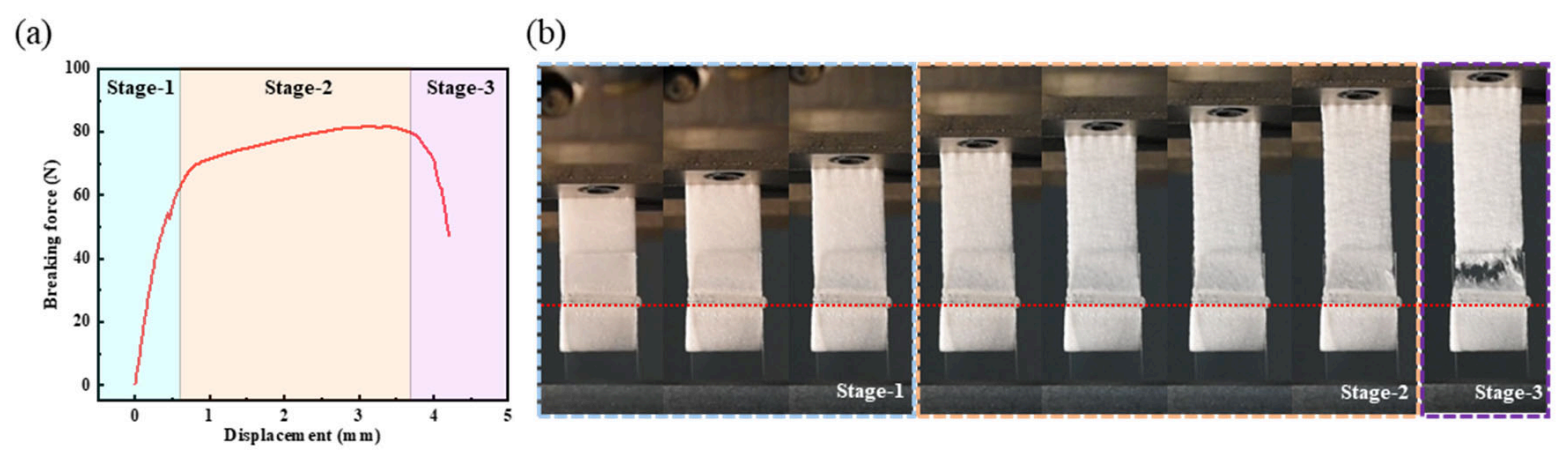
Mechanical performance and fracture process of the welded joint under recommended parameters. (**a**) The force–displacement curve. (**b**) Photographs of the tensile–shear process at different displacement stages, with color-coded regions consistent with (**a**).

Figure 9a shows the force–displacement curve obtained from the fracture shear test of the welded joint under the recommended parameters. The curve exhibits a steep linear rise in the elastic stage, followed by a yield plateau, and then gradually decreases after reaching the peak force, indicating a ductile fracture mode. The peak breaking force is 81.78 N, consistent with the predicted value. Three distinct stages can be identified from the force–displacement curve: Stage I (elastic–plastic deformation stage): The welded joint undergoes elastic and plastic deformation. The displacement increases slowly while the breaking force rises rapidly. Stage II (tensile tearing stage): The breaking force slowly increases to the peak, but the displacement increases significantly. The weld seam gradually tears as the deformation progresses. Stage III (fracture stage): The torn porous-PET substrate exhibits obvious plastic deformation and eventually fractures completely. Figure 9b shows the photographs of the tensile process corresponding to different displacement stages. The blue shaded area in the force–displacement curve (Figure 9a) corresponds to the photo marked by the blue dashed box in Figure 9b, and the stages marked by other colors follow the same pattern. As the displacement increases, the welded joint undergoes elastic deformation and yielding in sequence, and finally fractures at the porous-PET near the weld seam. It can be seen that the weld seam has high strength.

#### 4.4.3. Validation Experimental Results

Under the recommended parameter combination, three repeated welding experiments were conducted. The measured breaking forces, along with the original experimental data at 10 W and the predicted value at 10.55 W, are summarized in Table 10 for comparison. For the original dataset at 10 W (PET density 0.15 g/cm^3^, PC thickness 1.00 mm, welding speed 1.00 mm/s), the measured breaking forces were 81.00 N, 82.48 N, and 87.90 N. Although a single replicate reached 87.90 N, the other two values showed considerable deviation, indicating that this peak is likely an outlier due to experimental variability rather than a reliable representation of that parameter combination. The GPR model, which captures the overall trend of the response surface rather than memorizing individual outliers, predicted a breaking force of 81.78 N at 10 W. For the optimized condition at 10.55 W, the predicted breaking force was 82.26 N, and the rounded validation experiments at 11 W yielded consistent results (76.66 N, 81.78 N, and 80.10 N), with an average of 79.51 N and a standard deviation of 2.13 N. The model-predicted breaking force at 11 W was 81.67 N, yielding a relative error of 2.72%. The small prediction errors at both 10 W (2.40%) and 11 W (2.72%) confirm the good predictive capability of the GPR model and further demonstrate that the region around 10.55 W represents a favorable process window with stable and repeatable joint performance.

All three replicate samples exhibited consistent fracture behavior, with failure occurring within the porous-PET substrate rather than along the PC/porous-PET interface. These validation results demonstrate that the recommended condition provides stable and repeatable joint performance, supporting its use as a recommended process window. While three validation experiments at one condition provide only a preliminary verification, they confirm that the recommended process window yields stable and repeatable joint performance, supporting the feasibility of the proposed prediction–optimization framework. Extensive validation across multiple conditions is planned for future work.

Compared with previously reported LTW modeling studies that rely on neural networks [14,17], the proposed GPR-based framework offers a distinct advantage by retaining intra-group experimental variability through the use of full replicate-level data, which improves model generalization under small-sample conditions. This feature is particularly important when experimental data are limited and repeated measurements are available. Although GPR inherently provides uncertainty estimates, the current framework only takes the predicted mean value (breaking force) of GPR as the optimization objective; incorporating uncertainty information into the optimization loop is left as a promising direction for future work. From an engineering perspective, this framework is suitable for rapid process parameter screening in scenarios where constructing physical models is difficult or where experimental campaigns are costly. The current validation is limited to a single material combination and a narrow range of conditions; future work will extend the validation to broader parameter spaces and other material systems.

#### 4.4.4. Fracture Morphology Analysis

**Figure 10:**
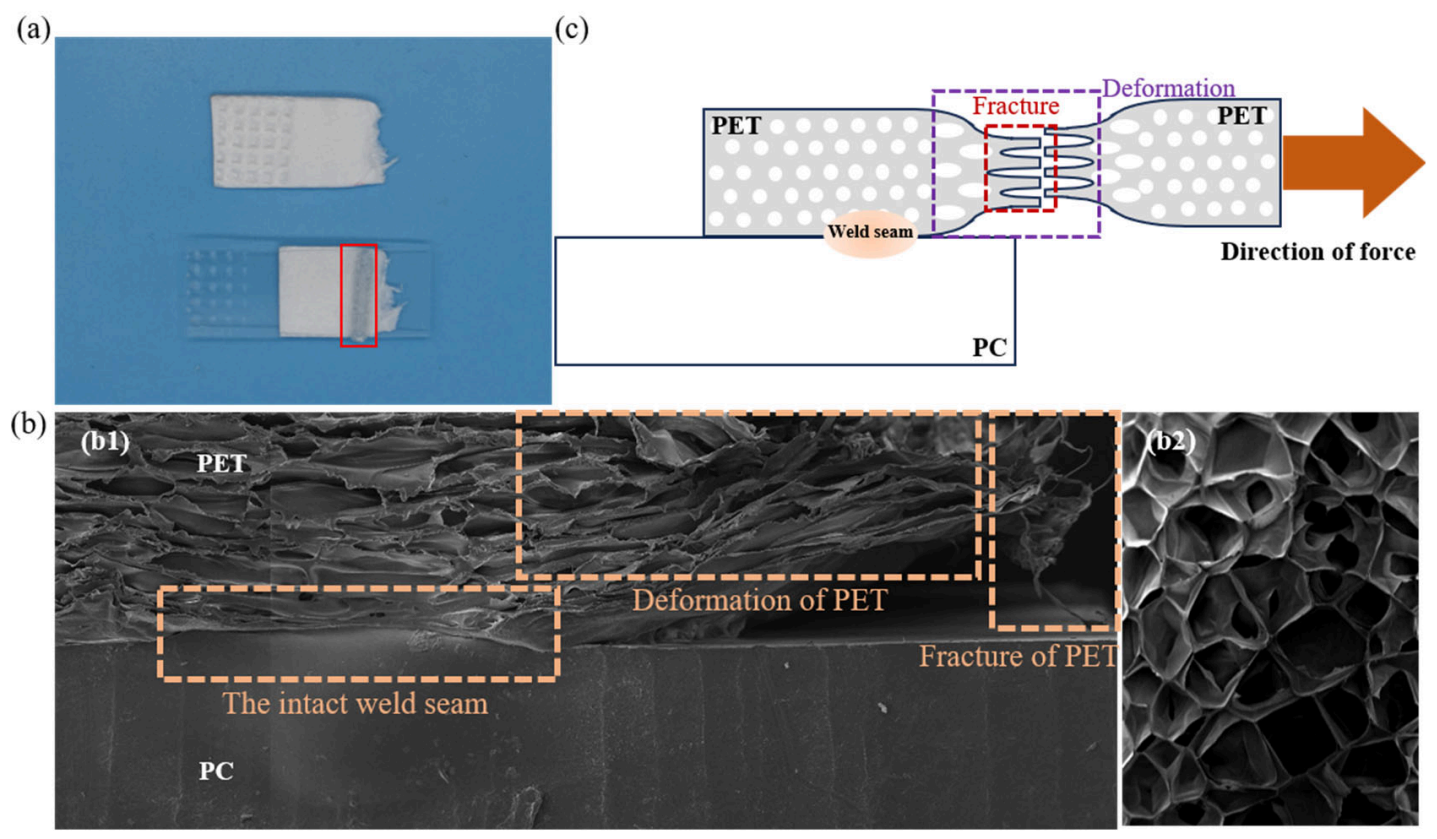
Fracture morphology and mechanism analysis of the welded joint under recommended parameters. (**a**) Macroscopic morphology of the specimen after fracture. (**b1**) SEM image of the fractured region: intact weld seam with distinct deformation and fracture zones on the porous-PET side. (**b2**) SEM image of the original porous-PET (0.15 g/cm^3^), showing highly irregular pores in shape and size. (**c**) Schematic of tensile fracture: stress concentrates at the weld edge, cracks initiate and propagate within the porous-PET, not along the PC/porous-PET interface.

To further investigate the fracture behavior after tensile failure and the integrity of the welded joint, the specimens after fracture were observed. Figure 10a shows the macroscopic morphology of the specimen after the tensile–shear test. It can be observed that fracture occurred on the porous-PET material side, while the PC plate remained intact, with no obvious delamination at the weld seam. Figure 10b1 presents a representative SEM image of the fractured surface. All three replicate samples showed consistent failure behavior, with the weld seam itself remaining almost intact and no obvious cracks or voids at the interface. On the porous-PET side, a large deformation zone and the fracture region of porous-PET can be clearly observed, indicating that the weld strength exceeds the cohesive strength of the porous-PET material. Figure 10b2 presents the SEM image of the original porous-PET (0.15 g/cm^3^), where the pores are highly irregular in shape and size. Compared with Figure 10b1, significant morphological changes in the pores can be observed after tensile deformation.

Based on the above observations, Figure 10c provides a schematic diagram of the tensile fracture mechanism, illustrating that under external force, stress concentrates at the edge of the weld seam. Cracks preferentially initiate and propagate within the porous-PET material, eventually leading to ductile fracture of the porous-PET, while the PC/porous-PET interface remains intact.

## 5. Conclusions

In this study, a forward prediction–reverse optimization strategy was successfully applied to identify a recommended process window for laser transmission welding of PC/porous-PET. The GPR model trained with the full experimental dataset achieved high predictive performance, with a test set R^2^ of 0.9201, and outperformed the GPR model trained with the averaged experimental dataset (test set R^2^ = 0.2517). Among the three optimization algorithms, CMA-ES demonstrated the fastest convergence (≈500 iterations) and the shortest runtime (2.69 s), while still converging to similar recommended parameters as GA and BO. The recommended parameters (porous-PET density 0.15 g/cm^3^, PC thickness 1.00 mm, welding speed 1.00 mm/s, welding power 11 W) were experimentally validated with a maximum relative error of 2.72%. Morphological analysis confirmed that the weld seam was uniformly formed with full fusion in the central region. While the proposed framework demonstrates promising predictive performance, the current study has several limitations. First, the identification of a recommended process window serves as a preliminary verification rather than a proven global optimum; extensive validation across multiple conditions is needed. Second, the physical mechanisms underlying the welding process—such as heat generation, melt flow, and molecular diffusion—have not been systematically investigated, as the primary focus of this work is on data-driven modeling and optimization. Future work will extend the experimental validation to broader parameter spaces and other material systems, and incorporate mechanistic analysis to further elucidate the process–structure–property relationships. The proposed strategy offers a promising and intelligent approach for precision welding in advanced manufacturing.

## Figures and Tables

**Table 1:** Specific values of experimental parameters.

Parameters	Unit	Values
Porous-PET density	g/cm^3^	0.15, 0.175
PC thickness	mm	0.3, 0.55, 1.0
Welding speed	mm/s	1, 1.5, 2, 2.5, 3, 4, 5
Welding power	W	4, 5, 6, 7.5, 8, 10, 12, 12.5, 15, 16, 20, 24

**Table 2:** The key parameters of GA.

Parameters	Values
Population size	50
Generations	300
Crossover rate	0.8
Mutation rate	0.15
Tournament size	3
Patience	30
Convergence eps	1 × 10^−6^

**Table 3:** The key parameters of BO.

Parameters	Values
Initial sample size	20
Number of iterations	80
Acquisition function restart count	20
Noise level	1 × 10^−6^

**Table 4:** Main parameters of CMA-ES.

Parameters	Values
Initial scale	0.25
Initial random sampling count	30
Number of iterations	600

**Table 5:** Cases for forward prediction and reverse optimization.

	Model & Algorithm	
**Forward prediction**	Proposed method	Experimental values
	Comparison case	Average values
**Reverse optimization**	Proposed method	CMA-ES
	Comparison case 1	GA
	Comparison case 2	BO

**Table 6:** GPR structure parameters under the full experimental dataset.

Parameters	Value Range	Optimal Value
Kernel	RBF, Matern, RQ	Matern
v	0.2, 0.5, 1.5, 2.5	0.5
Alpha	0.001, 0.01, 0.1, 1	0.01
N restarts optimizer	20	20
Length scale	To be optimized	2.15
Signal variance	To be optimized	1.01

**Table 7:** Comparison of prediction performance and single prediction time between ANN and GPR models over ten independent runs (normalized RMSE).

ANN	RMSE	R^2^	Runtime (ms)	GPR	RMSE	R^2^	Runtime (ms)
1	0.2664	0.9076	76.39	1	0.2477	0.9201	0.39
2	0.2571	0.9139	77.53	2	0.2477	0.9201	0.42
3	0.279	0.8986	73.46	3	0.2477	0.9201	0.42
4	0.2485	0.9195	73.32	4	0.2477	0.9201	0.40
5	0.2957	0.8861	69.12	5	0.2477	0.9201	0.47
6	0.2487	0.9194	77.91	6	0.2477	0.9201	0.42
7	0.2741	0.9021	72.82	7	0.2477	0.9201	0.41
8	0.2549	0.9153	74.05	8	0.2477	0.9201	0.47
9	0.2552	0.9152	69.68	9	0.2477	0.9201	0.45
10	0.284	0.8949	71.75	10	0.2477	0.9201	0.43
**AVG**	0.2665	0.9072	73.603	**AVG**	0.2477	0.9201	0.428

**Table 8:** Validation performance of the ANN and GPR model trained on the full experimental dataset across five independent data splits with different random seeds.

ANN	RMSE	R^2^	GPR	RMSE	R^2^
1	0.1989	0.9472	1	0.1977	0.9535
2	0.2510	0.9086	2	0.2134	0.9291
3	0.2861	0.8839	3	0.2492	0.9115
4	0.2363	0.9159	4	0.1996	0.9411
5	0.2145	0.9289	5	0.2014	0.9359
**AVG**	0.2374	0.9169	**AVG**	0.2123	0.9342

**Table 9:** Recommended parameter combinations and predicted performance of the three optimization algorithms.

Optimization Algorithm	Recommended Parameters				Predicted Breaking Force (N)	Runtime (s)
	Porous-PET Density (g/cm^3^)	PC Thickness (mm)	Welding Speed (mm/s)	Welding Power (W)		
CMA-ES	0.15	1.00	1.00	10.55	82.26	2.69
GA	0.15	1.00	1.00	10.54	82.26	6.32
BO	0.15	1.00	1.00	10.54	82.26	14.47

**Table 10:** Comparison of experimental and predicted breaking forces under different power conditions (PET density: 0.15 g/cm^3^, PC thickness: 1.00 mm, welding speed: 1.00 mm/s).

Case	Predicted Breaking Force (N)	Experimental Breaking Force (N)	Average Experimental Value (N)	Deviation (%)
P = 10 W V = 1 mm/s	81.78	81 82.48 87.9	83.79	2.40
P = 10.55 W V = 1 mm/s	82.26	—	—	—
P = 11 W V = 1 mm/s	81.67	76.66 81.78 80.10	79.51	2.72

## Data Availability

The original contributions presented in this study are included in the article. Further inquiries can be directed to the corresponding author.
